# Case report: Successful radical surgery following complete pathological remission of advanced HCC with Tislelizumab/Lenvatinib plus TACE

**DOI:** 10.3389/fonc.2024.1369567

**Published:** 2024-08-29

**Authors:** Yong Xie, Tianshi Lyu, Yinghua Zou, Jian Wang

**Affiliations:** Department of Interventional Radiology and Vascular Surgery, Peking University First Hospital, Beijing, China

**Keywords:** surgery, hepatocellular carcinoma, tislelizumab, lenvatinib, transarterial chemoembolization, case report

## Abstract

The combination therapy of Tislelizumab plus Lenvatinib has recently emerged as the new standard of care for unresectable hepatocellular carcinoma (HCC). This treatment has demonstrated a significant reduction in tumor burden, raising the possibility of conversion therapy. However, the full safety and efficacy of this combination in real-world settings are not yet fully understood. We recently reported the case of a 36-year-old man with initially unresectable massive HCC, for whom radical surgery (RS) was contraindicated. After receiving Tislelizumab/Lenvatinib plus transarterial chemoembolization (TACE), the patient achieved complete pathological remission and subsequently underwent RS. The patient did not experience postoperative severe complications, and there was no recurrence during the follow-up period. Tislelizumab/Lenvatinib plus TACE therapy may lead to a complete pathological response in advanced HCC. Nevertheless, the safety of prolonged treatment needs to be assessed.

## Introduction

Hepatocellular carcinoma (HCC) is a prevalent malignant tumor worldwide, with viral infection being the primary risk factor. Its incidence is steadily increasing, and additional factors such as alcohol consumption, obesity, and type 2 diabetes also contribute to its prevalence (1). Treatment options for HCC include surgical resection, liver transplantation, local ablation, embolization or radiation therapy, and systemic drug therapy (2–4). Advanced HCC is often complicated by macrovascular invasion and lacks radical methods, leading to the utilization of comprehensive treatment strategies (4, 5), so overall survival improvement is limited (6). Traditional therapies like transarterial chemoembolization (TACE) have shown efficacy in controlling tumor growth and prolonging survival, but often result in limited responses and disease progression. Therefore, there is an urgent need to search for more effective and safer treatments.

Targeted combined immunotherapy has emerged as a promising therapeutic strategy for HCC in recent years. This approach works by activating the patient’s immune system, enabling it to recognize and eliminate cancer cells more effectively. The emergence of immune checkpoint inhibitors, such as Tislelizumab, has revolutionized the treatment landscape for advanced HCC by enhancing anti-tumor immune responses and improving patient outcomes. Combining immunotherapy with targeted therapies like Lenvatinib has shown promising results in clinical trials, with increased response rates and prolonged progression-free survival compared to either therapy alone. However, the optimal sequencing and timing of these therapies in the context of complete pathological remission following treatment remain areas of ongoing research and clinical investigation. Compared to traditional treatments, targeted combined immunotherapy offers significant efficacy and mild side effects, leading to an improved quality of life and survival for HCC patients. However, targeted combination immunotherapy for HCC still faces challenges, such as individual differences in efficacy and immune-related side effects. In particular, the efficacy and safety of Lenvatinib plus Tislelizumab in the treatment of advanced HCC are still unclear (7–9).

Considering these aspects, herein, we report a case of TACE-combined Lenvatinib plus Tislelizumab administration to achieve conversion therapy for unresectable HCC.

## Case presentation

**Figure 1:**
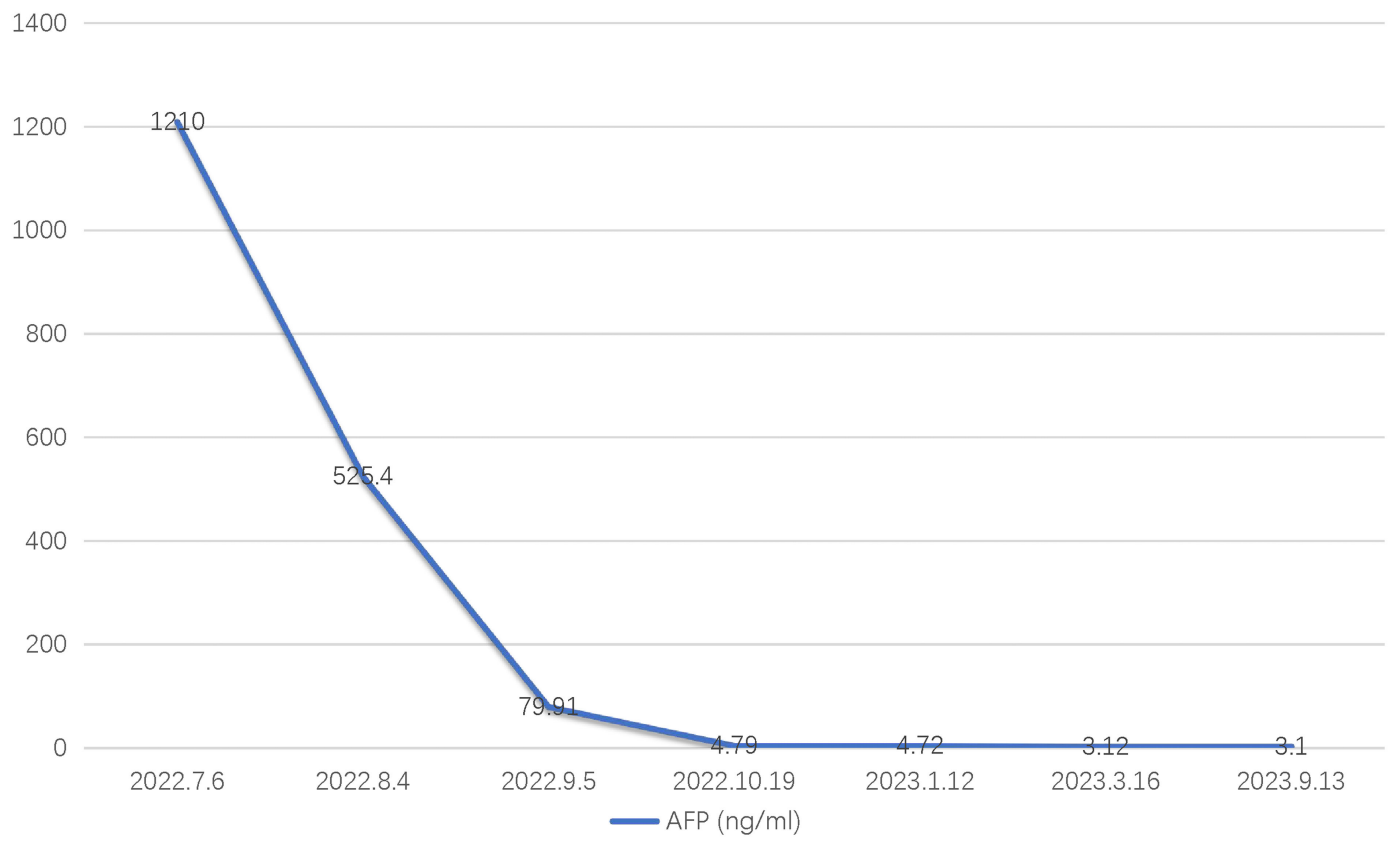
Dynamic change process from baseline AFP to September 2023.

**Figure 2:**
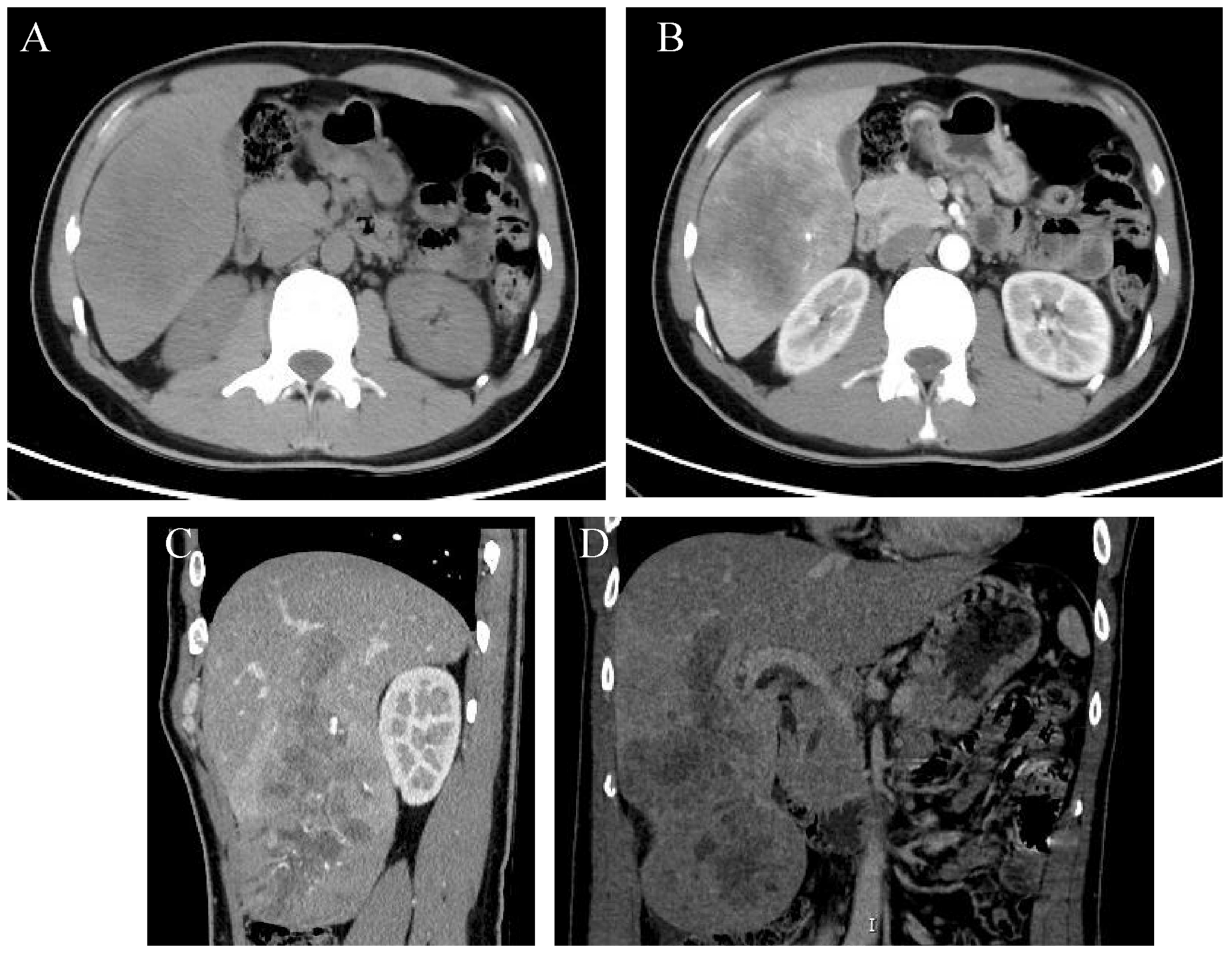
CT images showing a large mass in the right lobe of the liver, with the lesion invading the middle hepatic vein, measuring approximately 16.6×8.2 cm in size. **(A)** Non-enhanced. **(B)** Enhanced arterial phase. **(C, D)** Enhanced venous phase.

A 36-year-old male patient was referred to our center on July 6, 2022, with complaints of distension and pain in the right upper abdomen that had been present for 3 months. The patient had a history of chronic hepatitis B for one year (he regularly took entecavir tablets orally for a long time). In September 2021, he received antiviral therapy with tenofovir disoproxil fumarate and interferon. He denied a history of hypertension, diabetes, kidney disease, tuberculosis, and other infectious diseases. Physical examination showed a flat and soft abdomen with distension and pain in the right waist, no muscle tension or rebound pain, and mobile dullness (-). Subsequent laboratory analysis indicated that the patient tested positive for HBV, and the tumor marker alpha-fetoprotein (AFP) was significantly elevated at 1210 ng/ml (**Figure 1**). There were no obvious abnormalities in blood routine, stool routine, coagulation function, or liver and kidney function tests. In addition, contrast-enhanced computed tomography (CECT) showed a large mass in the right lobe of the liver (significant enhancement in the arterial phase and rapidly decreasing in enhancement in the venous phase) with multiple satellite foci, which had invaded the middle hepatic vein and was approximately 16.6 × 8.2 cm in size (**Figure 2**). Positron emission tomography/computed tomography (PET/CT) showed no evidence of systemic metastasis. Based on the results of physical examination, laboratory results, and imaging findings, the patient was diagnosed with advanced HCC (i.e., BCLC stage C) with a Child-Pugh class A (6 points), MELD-NA 4 score and Eastern Cooperative Oncology Group (ECOG) performance score 0 according to the updated Barcelona Liver Cancer staging system.

After discussions at the multidisciplinary tumor board, it was decided to administer TACE in combination with Lenvatinib and immunotherapy for conversion therapy. This approach aimed to inhibit tumor growth and promote compensatory hyperplasia in the left liver. Subsequently, the patient underwent a liver biopsy and TACE, followed by treatment with Lenvatinib (8mg once daily) and Tislelizumab (200mg every 3 weeks) on the first and fourth day after the operation, respectively.

**Figure 3:**
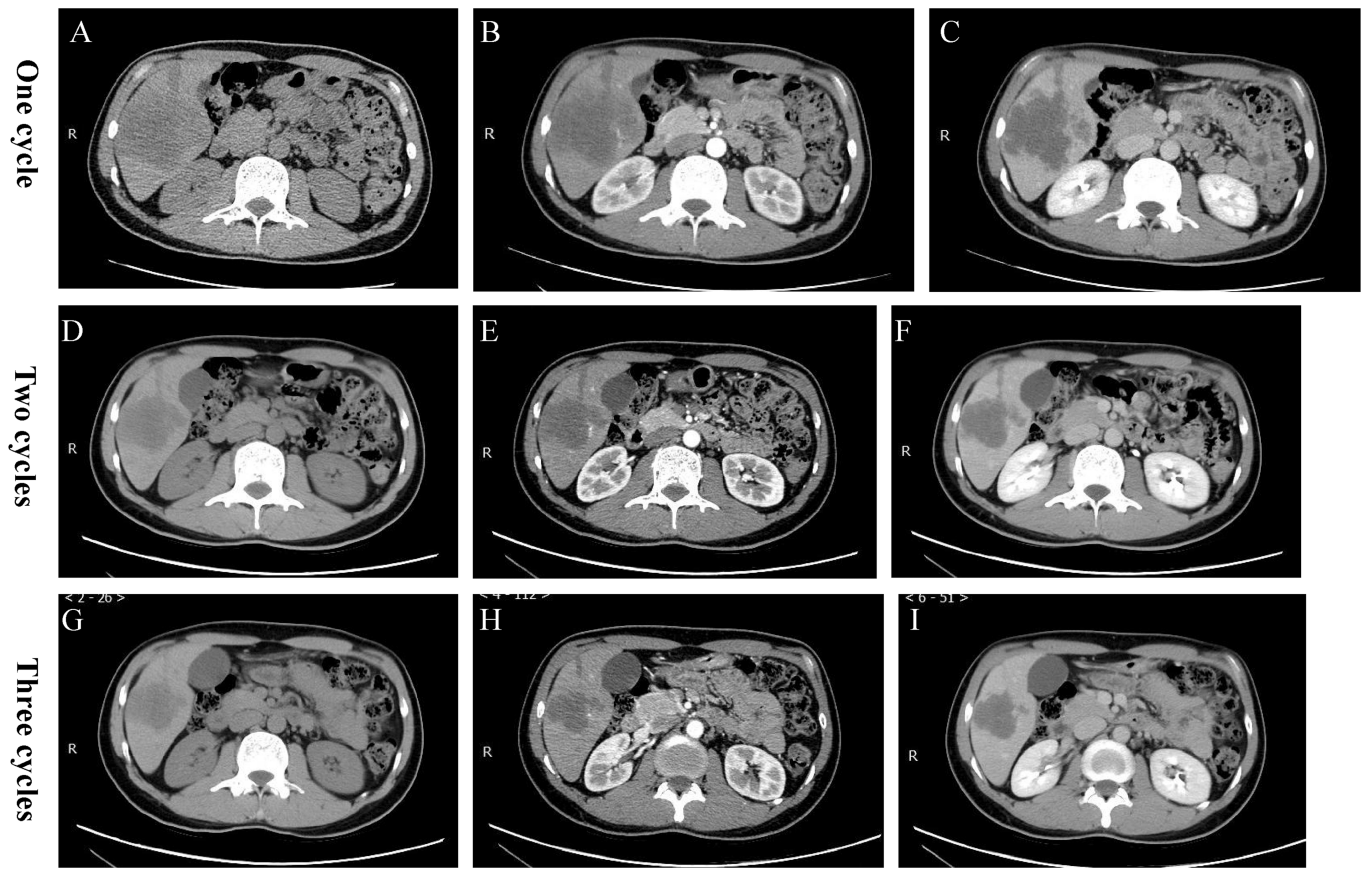
Postoperative CT images. **(A-C)** After undergoing one cycle of TACE plus Tislelizumab/Lenvatinib, CT showed a reduction in the size of the enhanced extent of the massive lesion in the right lobe of the liver, with an increase in necrotic components compared to before. **(D-F)** After undergoing two cycles of TACE plus Tislelizumab/Lenvatinib, there was a further increase in the necrotic components within the lesion. **(G-I)** After undergoing three cycles of TACE plus Tislelizumab/Lenvatinib, the degree of necrosis met the PR criteria of the RECIST standard.

Two weeks later, on July 29, 2022, after the operation, subsequent contrast-enhanced computed tomography (CECT) scans showed a decrease in the size of the liver mass and an increase in necrotic components within the lesion (**Figure 3**). On August 4, 2022, the patient underwent TACE for the second time due to tumor staining observed in hepatic arteriography. One month after the second TACE, on September 6, 2022, the CECT scan revealed uneven lipiodol deposition in the large HCC in the right lobe of the liver, with the lesion showing a decrease in size compared to before, and the enhancement was not obvious (**Figure 3**). Subsequently, on September 8, 2022, the patient underwent the third TACE. On October 20, 2022, a follow-up CECT scan showed that the HCC lesions did not exhibit enhancement, with large necrotic areas visible during the imaging period (**Figure 3**). After discussion at the multidisciplinary tumor board, it was agreed that a combination of local and systemic treatments could reduce the tumor stage, shrink the tumor, and potentially allow for radical hepatectomy. On October 28, 2022, the patient successfully underwent liver surgery. Pathological examination revealed Edmonson-Steiner grade III (WHO grade: poorly differentiated), with no evidence of microvascular invasion and no viable tumor cells. In addition, the tumor did not invade the liver capsule, and there was no sign of nerve and lymph node invasion. Immunohistochemistry results demonstrated Hepatocyte (-), Arginae1(+), GPC3 (foci+), AFP(-), CK19(++), CK7 (5+), CD34 (vascularization), CD10(-), p53(+), and Ki67 (20%), with TTF1 (-).

**Figure 4:**
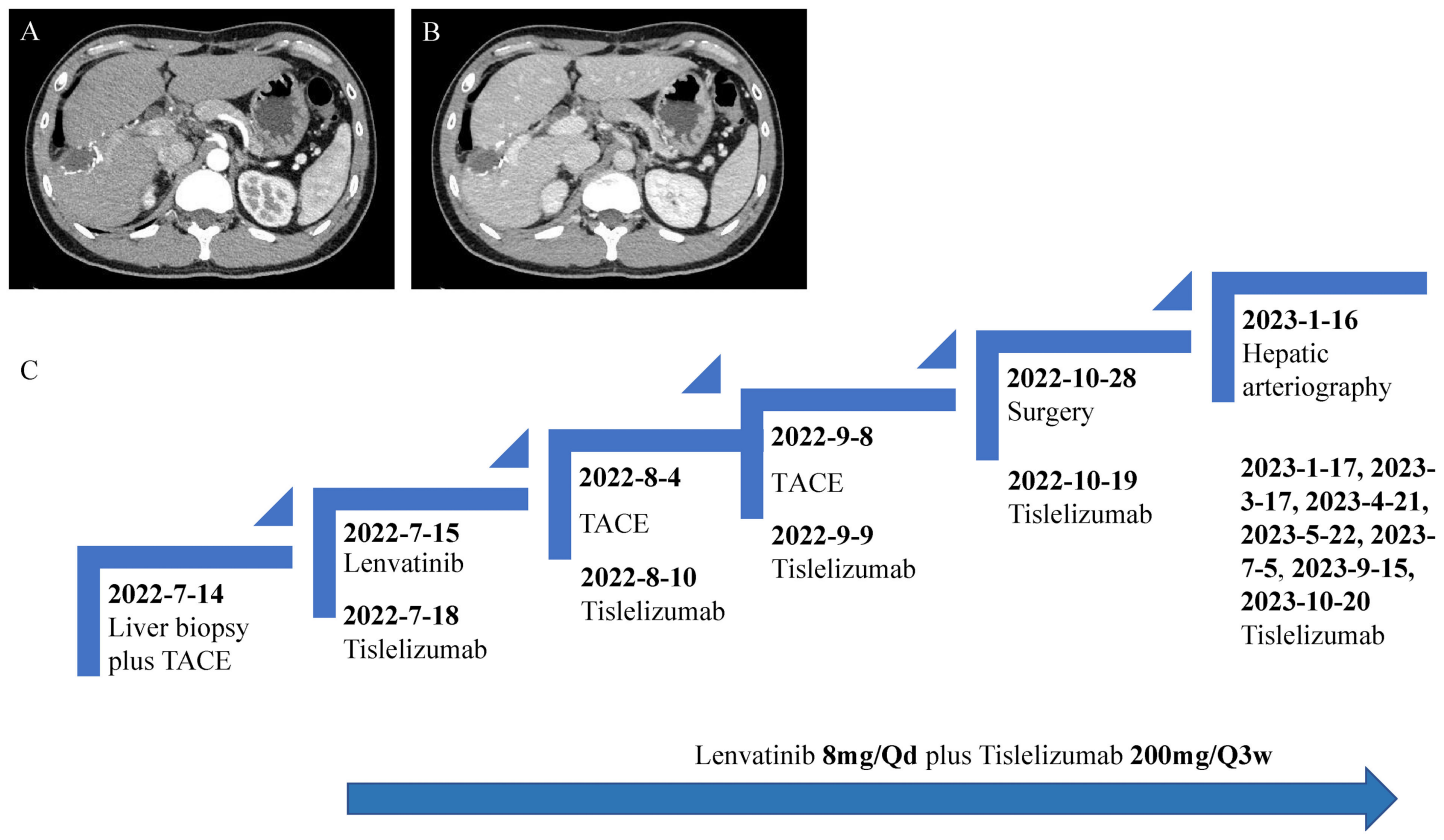
**(A, B)** As of October 2023, CT scan revealed that there has been no tumor recurrence. **(C)** The patient’s treatment process.

After the surgery, the patient attended regular follow-up appointments at our hospital. On January 16, 2023, hepatic arteriography showed no evidence of tumor staining. Subsequent CECT and magnetic resonance imaging (MRI) on March 16, 2023 also revealed no obvious abnormal enhancement of the original lesion, indicating that no signs of tumor recurrence were found. Follow-up appointments, including hepatic arteriography and CECT or MRI scans, were conducted until October 20, 2023, all of which indicated no tumor recurrence (**Figures 4A, B**). The patient experienced a grade 1 decrease in appetite as a treatment-emergent adverse event. This was managed with symptomatic drug treatment and gradually resolved without any severe adverse reactions. A detailed overview of the patient’s treatment process is outlined in **Figure 4C**.

## Discussion and conclusion

Here, we present a case report of a patient with HCC who was initially ineligible for curative options. The patient was successfully treated with Tislelizumab/Lenvatinib plus TACE, leading to complete tumor disappearance and subsequent RS. Importantly, the patient has shown no signs of tumor recurrence to date.

The patient presented with advanced HCC with hepatic vein invasion, placing them at clinical stage BCLC stage C. Due to insufficient remaining liver volume, surgical treatment was not recommended. Instead, systemic drug treatment was advised, with the possibility of reevaluation for surgery after transformation and downstaging. Targeted combination immunotherapy for HCC has been a focus of extensive research, with significant advancements made in recent years (10–13). HCC is a molecularly heterogeneous tumor involving processes such as neovascularization, changes in the microenvironment, uncontrolled cell division and proliferation, and suppression of the immune system. Therefore, targeted therapy and immunotherapy play a crucial role in its treatment.

Multi-targeted tyrosine kinase inhibitors (TKIs) have now become the new standard of care for patients with advanced HCC. Agents such as sorafenib, lenvatinib, donafenib, regorafenib, and cabozantinib have demonstrated positive effects in inhibiting tumor proliferation, neovascularization, metastasis, and anti-apoptosis. However, single-targeted molecularly targeted drugs have shown a low short-term effectiveness rate, with most patients surviving for approximately one year, thus rendering the overall efficacy unsatisfactory. Regarding immunotherapy, PD-1/L1 inhibitor monotherapy has not shown ideal efficacy in HCC patients. Tislelizumab is a monoclonal antibody targeting PD-1, while Lenvatinib is a TKI with anti-angiogenic activity. The combination of these two agents has displayed promising results in the treatment of advanced HCC, with an overall response rate of 41% and a manageable safety profile in a phase 1b/2 trial. On the other hand, TACE is a well-established locoregional therapy for HCC, delivering chemotherapy directly to the tumor and inducing ischemic necrosis through the embolization of the feeding vessels. Combination therapy with targeted or immune therapy has emerged as the primary strategy. Immunotherapy targets the broad-spectrum properties of tumors and enhances treatment outcomes by modulating factors in the immune circulation. Although the average response rate is approximately 20%, patients can achieve relatively long survival once an effective response is achieved.

Currently, molecular targeted therapy combined with immunotherapy has become an important method for conversion therapy in advanced HCC. Many studies suggest that the conversion rate of combination therapy is significantly higher than that of monotherapy (14, 15). According to the Chinese expert consensus on HCC conversion therapy, it is recommended to combine various treatment methods to improve the success rate of conversion (16). In the case of our patient, who had a tumor in the right lobe of the liver and good liver function (Child-Pugh grade A6) with no contraindications to TACE therapy, conversion therapy was carried out using Tislelizumab/Lenvatinib plus TACE. The patient achieved partial remission based on RECIST criteria after multiple cycles, with the maximum tumor diameter reducing from 16.6 cm to 7.5 cm. Although the clinical stage did not decrease, the tumor burden was significantly reduced, and the patient met the criteria for resectability due to their good general condition (ECOG score of 0), good laboratory findings (such as liver reserve, etc) (**Supplementary Material**), and no clear evidence of extrahepatic metastasis. This suggests the potential synergistic effect of combining systemic immunotherapy with locoregional therapy in HCC. The immunomodulatory effect of Tislelizumab may enhance the anti-tumor immune response, while Lenvatinib’s anti-angiogenic properties can modulate the tumor microenvironment, making it more susceptible to TACE-induced cytotoxicity. Subsequently, after radical surgery, R0 resection was achieved, confirming the effectiveness of conversion therapy pathologically. The patient remained recurrence-free for 1 year after surgery, benefiting from his long-term survival. A noteworthy improvement in median overall survival was observed in a recent study by Wang et al. (7), indicating that the PD-1-Lenvatinib-TACE regimen may have an advantage over Lenvatinib-TACE (26.8 vs 14.4 months; P = 0.027). Additionally, the median progression-free survival (PFS) time differed between the two treatment groups, with 11.7 months in the PD-1-Lenvatinib-TACE group compared to 8.5 months in the Lenvatinib-TACE group (P = 0.028). Tan et al. (17) demonstrated that, according to RECIST 1.1 criteria, tumor shrinkage was observed in all patients, with an objective response rate of 61.1% (11/18) and a disease control rate of 94.4% (17/18), consistent with a previous study (18). Successful conversion was noted in 61.1% (11/18) of patients. As of the cutoff date, the actual pathological complete response rate was 57.1% (4/7); all seven patients underwent hepatectomy and recovered well post-surgery. A recent cohort by Chen et al. (9) included the quadruple (hepatic arterial infusion chemotherapy plus Lenvatinib and Tislelizumab with TAE, THRP) and the triple (hepatic arterial infusion chemotherapy plus Lenvatinib and Tislelizumab, HLP) with a total of 100 patients. The ORR of the THRP group was significantly better than that of the THRP group (72% vs 52%, P=0.039). OS (median, 14.1 versus 11.3 months, P=0.041) and PFS (median, 5.6 versus 4.4 months, P=0.037) were significantly longer in the THRP group compared with the HLP group. The safety profile of the two groups was comparable. Similarly, the latest study (19) found that Tislelizumab in combination with Lenvatinib showed good anti-tumor activity and good tolerability as a first-line treatment for patients with HCC, providing an alternative first-line treatment option for the treatment of HCC. However, due to the limitations of sample size, single-arm design, and short follow-up time, its role still needs to be verified by further prospective bulk studies. These results suggest that the combination therapy significantly extended survival time in patients with HCC, without any unexpected safety-related complications (7–9, 17–19). In addition, Tislelizumab in combination with Lenvatinib has made significant progress in the field of neoadjuvant therapy. At the 2023 ASCO meeting, preliminary clinical studies (20) showed that Tislelizumab combined with Lenvatinib in neoadjuvant therapy can significantly improve the objective response rate of tumors, providing patients with more treatment options. At the same time, the safety profile of this combination therapy is relatively good and the patient tolerability is high, which provides strong support for clinical practice. In the future, this therapy is expected to play an important role in the neoadjuvant treatment of more tumor types, bringing better treatment outcomes to patients. Our case differs from the above studies in that not only did we achieve good results (9, 19), but also achieved successful conversion rather than neoadjuvant effects (20). Quality of life is a crucial consideration in cancer treatment, reflecting patients’ overall well-being and satisfaction during therapy. In this study, improvements were observed in various quality of life factors including physical functioning, emotional well-being, social functioning, and overall health perception, indicating that the treatment regimen not only targets the disease but also positively impacts patients’ daily functioning and psychological well-being. The combination of Tislelizumab and Lenvatinib in conjunction with TACE may offer a synergistic effect in controlling tumor progression and improving overall survival, ultimately enhancing the quality of life for patients. The use of Tislelizumab, Lenvatinib, and TACE in treating advanced HCC demonstrates potential benefits beyond disease control, also improving patients’ quality of life. Future prospective studies with larger sample sizes and longer follow-up periods are needed to further assess the impact of this treatment regimen on quality of life outcomes and validate these findings. Altogether, enhancing the quality of life for HCC patients should be an essential component of cancer treatment strategies, ensuring comprehensive care and patient-centered outcomes.

In recent years, the prognosis of patients with HCC has significantly improved, primarily due to advancements in surgical technology and the development of anti-tumor drugs, along with the adoption of multi-modal combination therapy (4–7, 9, 14, 15, 17–21). Intermediate or advanced stages of HCC have shifted from a lack of effective drugs to the continuous emergence of targeted drugs, paving the way for radical surgery. Patients with HCC combined with venous invasion encounter a poor overall prognosis, are prone to intrahepatic or extrahepatic metastases, and face a high postoperative recurrence rate. The diagnosis and treatment process of these patients has shown that the combined use of tyrosine kinase inhibitors and immune checkpoint inhibitors, in addition to locoregional therapy, can improve the clinical outcomes for this population and may serve as a viable option for conversion therapy. However, the effectiveness of various systemic drugs (4–7, 9, 14, 15) needs validation through several prospective clinical studies to identify appropriate populations for each treatment. Several challenges and considerations arise from this treatment approach. Firstly, the optimal timing and sequencing of TACE in relation to systemic therapy require careful evaluation to maximize therapeutic efficacy while minimizing adverse events. Secondly, the selection of patients who would benefit the most from this combination therapy remains a subject of ongoing research, as not all patients may respond favorably or experience manageable toxicities. Additionally, addressing the long-term outcomes and the potential for disease progression after treatment discontinuation are important considerations.

The introduction of novel immunotherapy agents such as Tislelizumab in combination with targeted therapy like Lenvatinib has shown promising results in the treatment of advanced HCC. However, the high cost of these agents and the additional cost of administering TACE procedures must be considered in the overall management of advanced HCC. The cost implications of using Tislelizumab/Lenvatinib plus TACE in the treatment of advanced HCC may include the expense of the drugs themselves, the cost of administering TACE procedures, hospitalization costs, monitoring and follow-up costs, and potential adverse events management costs. These expenses can significantly impact the overall healthcare budget for patients and healthcare systems. It is important for policymakers, healthcare providers, and payers to consider the cost-effectiveness of this treatment approach in comparison to the traditional standard of care for advanced HCC. Further research is needed to evaluate the cost-effectiveness of Tislelizumab/Lenvatinib plus TACE and to explore potential strategies to mitigate the financial burden associated with this treatment regimen.

In conclusion, the case report of advanced HCC treated with the combination of Tislelizumab and Lenvatinib, along with TACE therapy, may lead to a complete pathological response and offer valuable insights into the evolving landscape of HCC therapy. The integration of systemic immunotherapy with locoregional therapy shows promise for improving treatment outcomes in this challenging disease. Further clinical trials and real-world evidence are needed to validate the efficacy and safety of this combination and to determine the optimal patient population for whom this approach may be most beneficial.

## Data Availability

The original contributions presented in the study are included in the article/**Supplementary Material**. Further inquiries can be directed to the corresponding author.
