# Supplementary material for: Case report: Successful radical surgery following complete pathological remission of advanced HCC with Tislelizumab/Lenvatinib plus TACE
Source: Front Oncol. 2024 Aug 29;14:1369567. doi: 10.3389/fonc.2024.1369567 (PMC11390427; doi:10.3389/fonc.2024.1369567)

**[Supplementary material](https://www.frontiersin.org/journals/oncology/articles/10.3389/fonc.2024.1376270/full#hsm)**

The changing trends of these indicators (liver function parameters, complete blood count and coagulation function parameters, etc.) during the treatment. ALT, alanine aminotransferase; AST, aspartate aminotransferase; ALB, serum albumin; RBC, red blood cell count; Hb, haemoglobin; PLT, platelet count; PT, prothrombin time.


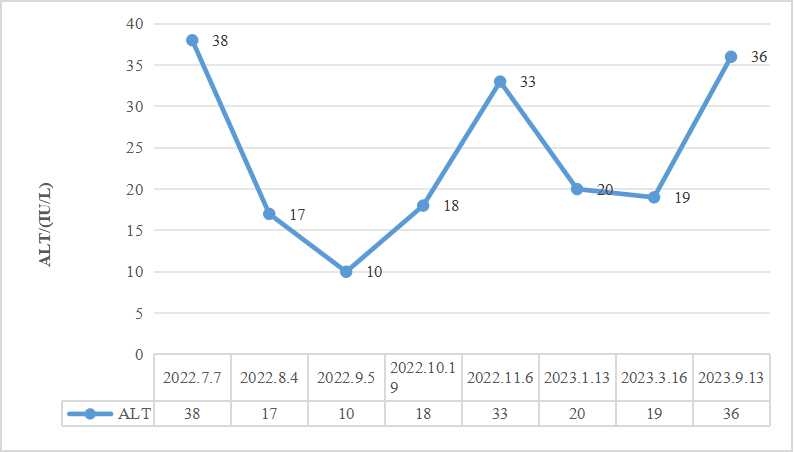


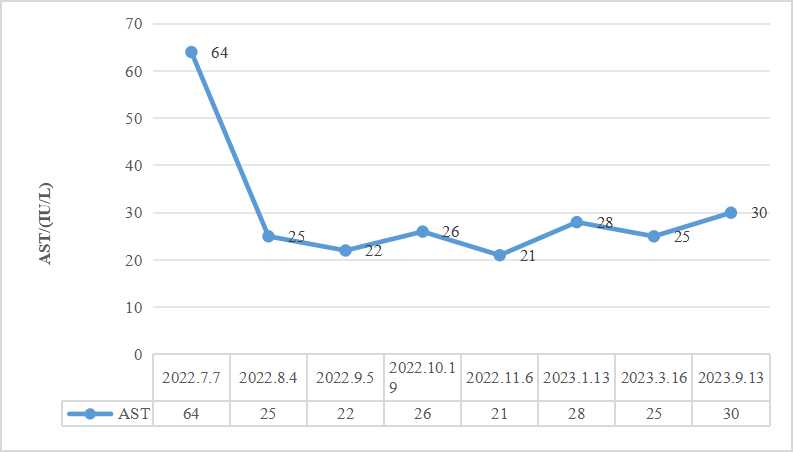


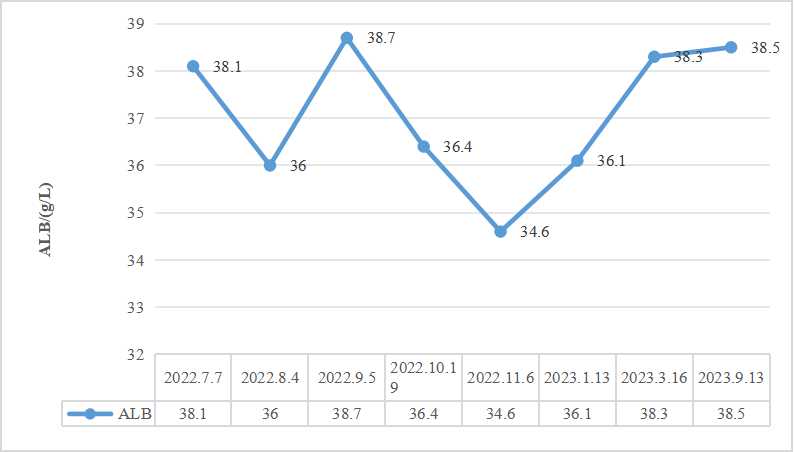


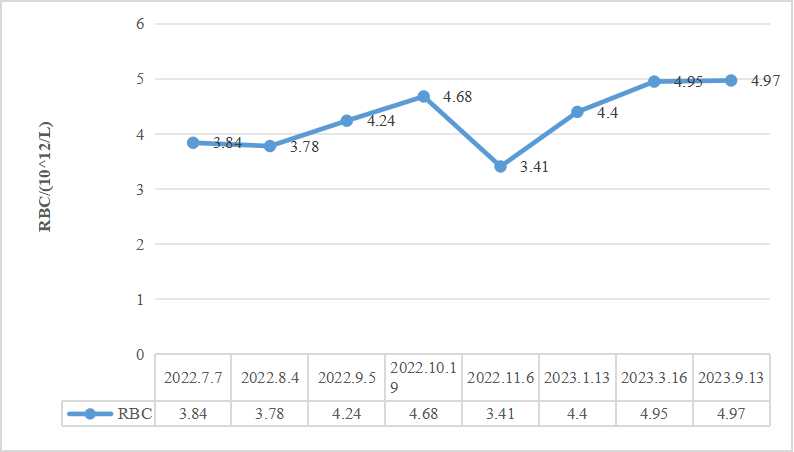


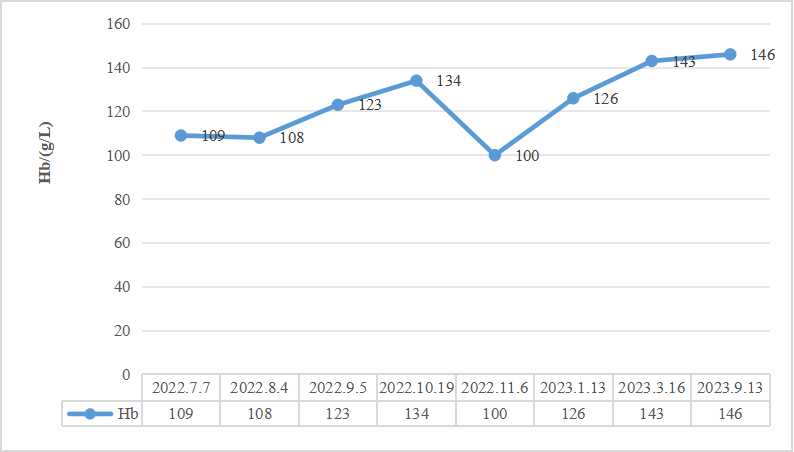


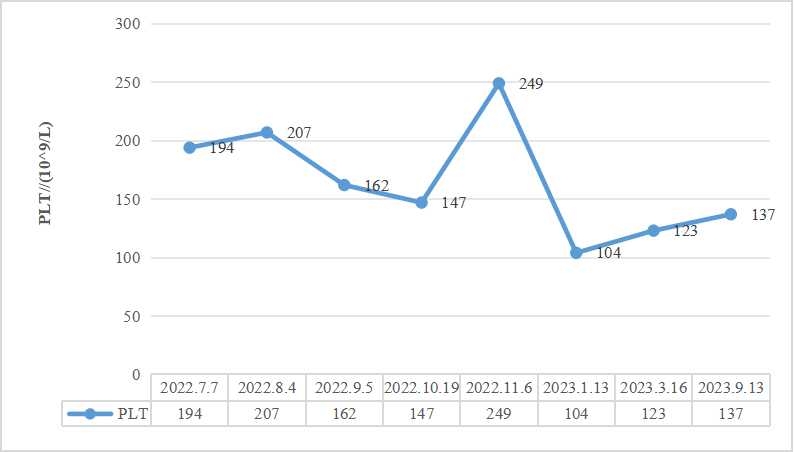


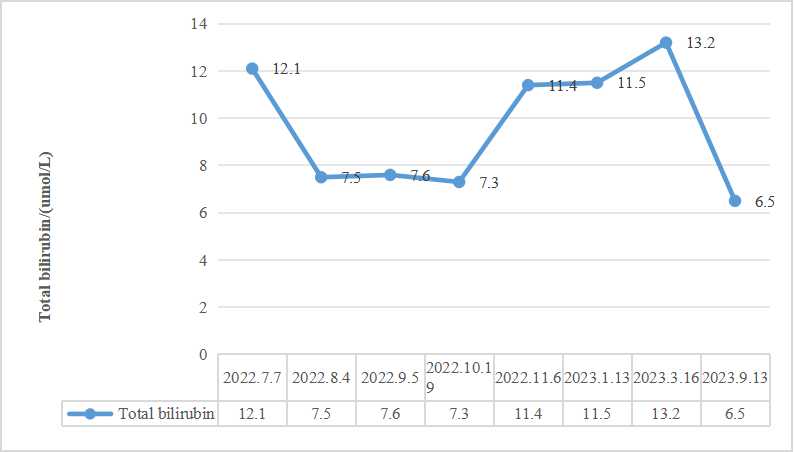


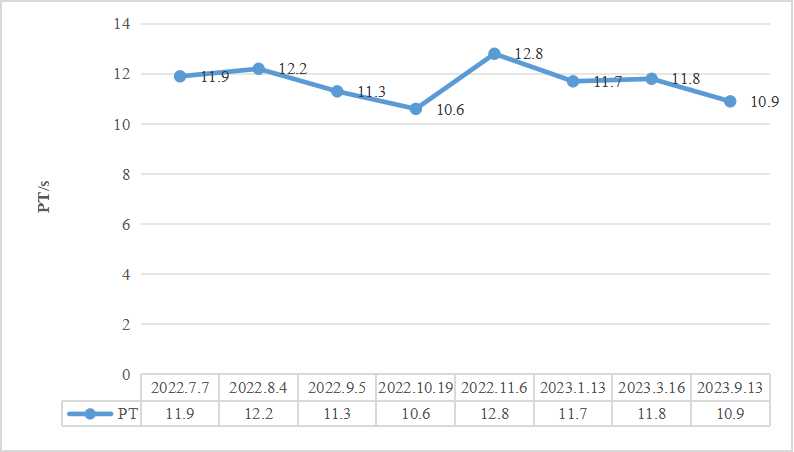

Supplement: Supplementary file 1 [file DataSheet1.docx]
